# Management outcomes in pubic diastasis: our experience with 19 patients

**DOI:** 10.1186/1749-799X-6-21

**Published:** 2011-05-17

**Authors:** Sameer Aggarwal, Kamal Bali, Vibhu Krishnan, Vishal Kumar, Dharm Meena, Ramesh K Sen

**Affiliations:** 1 Dept of Orthopaedics, PGIMER, Chandigarh, Postgraduate Institute of Medical Education and Research, Sector 12, Chandigarh - 160 012, India

## Abstract

**Background:**

Pubic diastasis, a result of high energy antero-posterior compression (APC) injury, has been managed based on the Young and Burguess classification system. The mode of fixation in APC II injury has, however, been a subject of controversy and some authors have proposed a need to address the issue of partial breach of the posterior pelvic ring elements in these injuries.

**Methods:**

The study included a total of 19 patients with pubic diastasis managed by us from May 2006 to December 2007. There was a single patient with type I APC injury who treated conservatively. Type II APC injuries (13 patients) were treated surgically with symphyseal plating using single anterior/superior plates or double perpendicularly placed plates. Type III injuries (5 patients) in addition underwent posterior fixation using plates or percutaneous sacro-iliac screws. The outcome was assessed clinically (Majeed score) and radiologically.

**Results:**

The mean follow-up was for 2.9 years (6 months to 4.5 years). Among the 13 patients with APC II injuries, the clinical scores were excellent in one (7.6%), good in 6 (46.15%), fair in 4 (30.76%) and poor in 2 (15.38%). Radiological scores were excellent in 2 (15.38%), good in 8 (61.53%), fair in 2 (15.38%) and poor in one patient (7.6%). Among the 5 patients with APC III injuries, there were 2 patients each with good (50%) and fair (50%) clinical scores while one patient was lost on long term follow up. The radiological outcomes were also similar in these. Complications included implant failure in 3 patients, postoperative infection in 2 patients, deep venous thrombosis in one patient and bladder herniation in one of the patients with implant failure.

**Conclusions:**

There is no observed dissimilarity in outcomes between isolated anterior and combined symphyseal (perpendicular) plating techniques in APC II injuries. Single anterior symphyseal plating along with posterior stabilisation provides a stable fixation in type III APC injuries. Limited dissection ensuring adequate intactness of rectus sheath is important to avoid long term post-operative complications.

## Background

The fractures of the pelvic ring have been reckoned by orthopedicians, for long, as annihilating injuries with resultant high mortalities. Various classification systems have been proposed by different authors over the years, in an attempt to create a better understanding of the biomechanics of this trauma and to devise proper management protocols for these high velocity injuries[1].

Diastasis of the pubic symphyseal joint has been reported to occur in 13 - 16% of pelvic ring injuries and it typically follows a very high velocity force with predominant external rotatory vector trying to split open one or both the hemipelvis. These injuries have been also been associated with various other situations like pregnancy, inflammatory arthritis following long-term corticosteroid intake, horse riding injuries etc. and carry high rates of complications and mortalities [2-4].

In the present article, we discuss our experience with patients who presented to us with similar injuries. We also try to highlight upon the associated injuries observed, the management protocols implemented, the fixation modalities employed and the complications encountered by us during management of these cases.

## Materials and methods

The study included a total of 19 patients with pubic diastases without any associated acetabular injuries admitted at the emergency orthopedic services of our hospital during the period May 2006 to December 2007. The vital parameters and the hemodynamic status of all patients were evaluated at admission and adequate resuscitation with fluids and blood transfusions carried out. A primary surveillance was carried out at the emergency ward in all these patients and all the other associated injuries were treated simultaneously by the concerned specialists. The patients were included in the study after obtaining written, informed consent.

In all the patients, standard pelvic roentgenograms, including antero-posterior (AP), inlet and outlet views of the pelvis and the Judet views for the evaluation of acetabulum were carried out; followed by computerized tomography (CT) scans. After adequate stabilization of the general condition of the patients, they were planned and taken up for appropriate surgical interventions. Patients with open injuries or persistent hypotension were initially stabilized with external fixators and a delayed open reduction and internal fixation procedure was carried out as early as their general condition allowed. All other patients underwent primary open reduction with internal fixation.

The pelvic injuries were assessed and classified as suggested by Young and Burguess [5]. The patients with type I APC injury were treated conservatively. Type II APC injuries were treated surgically with symphyseal plating using single anterior/superior plates or double plating with perpendicularly placed anterior and superior symphyseal plates (each plate fixed using two screws in each hemi pelvis). The choice of single or double plating in the Type II injury group depended upon the surgeon's preference. Type III injuries had fixation of the posterior using symphyseal plates or percutaneous sacro-iliac screws in addition to the anterior fixation using symphyseal plating. We used double plating for symphysis for only one of our patients with Type III injury; the rest of the patients were stabilized anteriorly using a single symphyseal plate.

### Surgical technique

**Figure 1:**
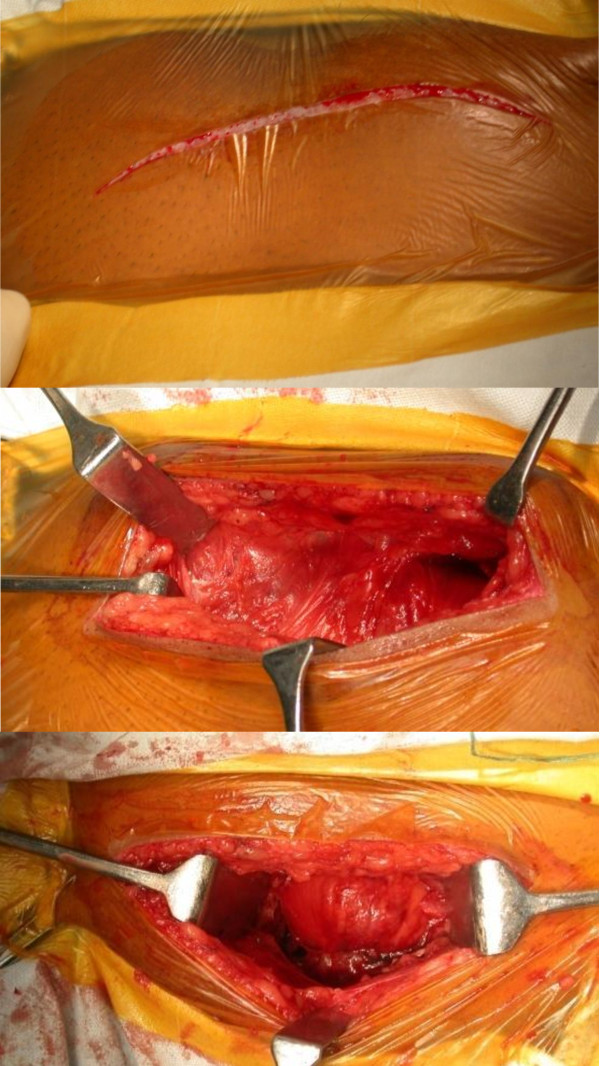
**Surgical Approach. Draping of the patient from 2 fingers below the pubis symphysis to 2 fingers superior to the umbilicus and a transverse Pfannenstiel incision (7-12 cms) being used**.

**Figure 2:**
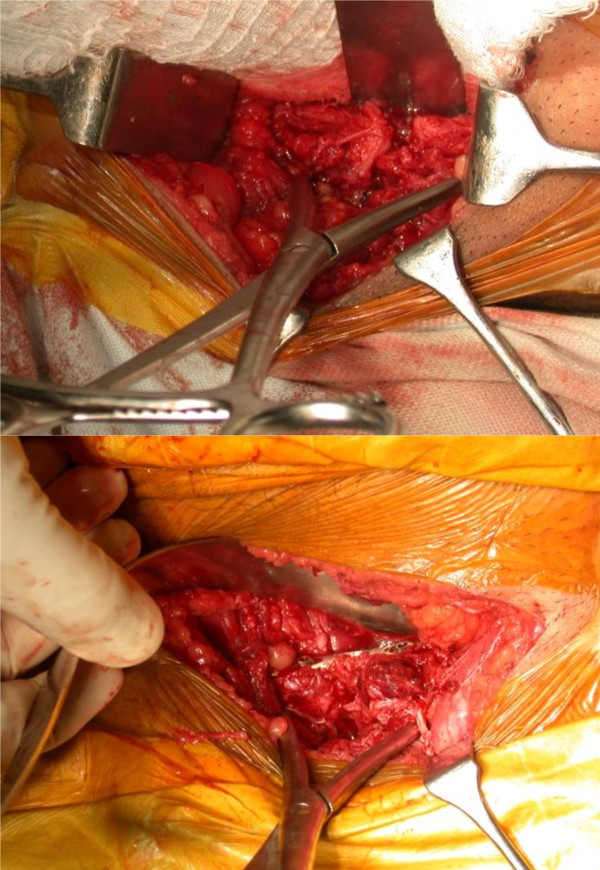
**Symphysis displacement reduction maneuver; placement of large pointed pelvic reduction clamps on each side of the symphysis and superior placement of plate in this case to maintain reduction**.

**Figure 3:**
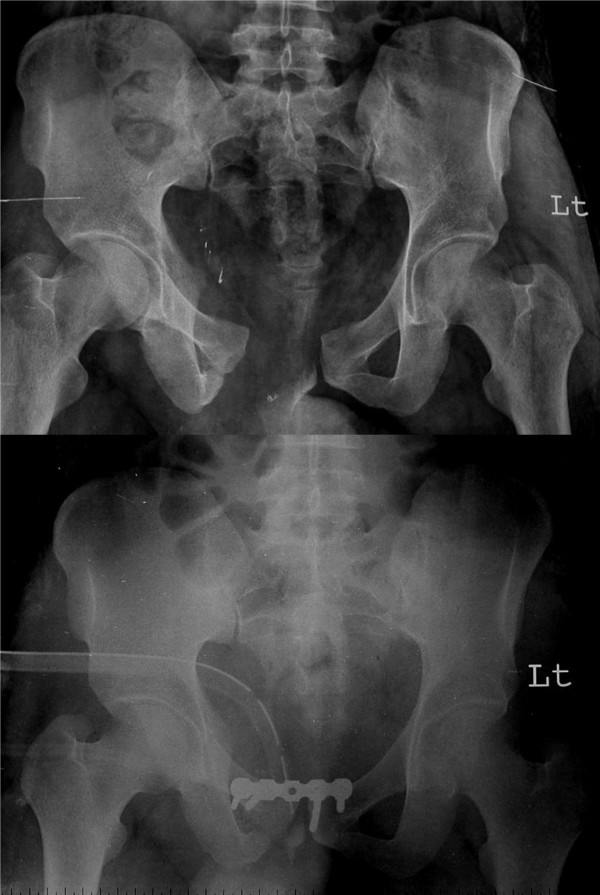
**Symphyseal fixation using single plating**.

**Figure 4:**
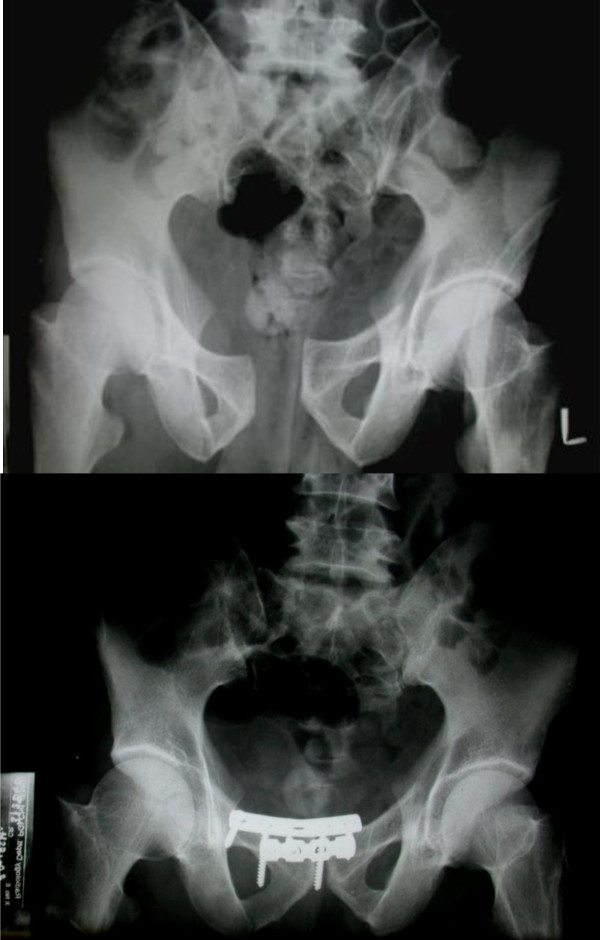
**Symphyseal fixation using double plating**.

The draping of the patient was from 2 fingers below the pubis symphysis to 2 fingers superior to the umbilicus. A transverse Pfannensteil incision, typically 7 - 12 cm long, was used exposing the anterior abdominal wall with the strong fascia of rectus muscle (Figure 1). In severe APC injuries, one head of rectus abdominis muscle might be avulsed. Linea alba was divided anteriorly in the midline, with the elevation of abdominis muscle at its insertion laterally. Transverse resection of the rectus abdominis muscle should be avoided (as this would impair further healing and repair of the abdominal wall). The reduction was usually achieved using a pointed reduction forceps or the pelvic reduction clamp (after the insertion of screws) (Figure 2). The fixation was achieved in our cases using an anterior or superior symphyseal plate (3.5 mm Low Contact Dynamic Compression Plates) (Figure 3) or double plating method (3.5 mm Low Contact Dynamic Compression Plates superiorly and a 3.5 mm reconstruction plate anteriorly) (Figure 4). A posterior plate/iliosacral screw was added in cases of Type III APC injuries.

### Post-operative protocol

The patients were maintained on post-operative prophylactic intravenous antibiotics for the initial 24 hours. In all patients, physical therapy was begun on the first post-operative day. Active hip, knee and ankle movements were encouraged. The patients with APC II injuries were instructed to commence touch-down weight bearing (immediately in the post-operative period) using crutches, or walker as assistive devices, followed by partial, progressive weight bearing at the end of 6 post-operative weeks. Unrestricted weight bearing on the ipsilateral limb was commenced after the completion of 3 months. In the APC III injuries, the rehabilitation protocol was different, with a more delayed commencement of progressive, partial weight bearing on the affected limb (not earlier than 3 months post-operatively). Thromboprophylaxis with low molecular heparin was administered in all patients post-operatively for 10 days. Any complication was identified and adequately treated. The patients were discharged on the 14^th ^post-operative day after the removal of sutures (except in cases where the post-operative complications warranted a longer duration of hospital stay).

The patients were followed up 6 weekly for the first 6 months, every 3 months after that until a year and thereafter once a year. The patients were assessed clinically during each visit and the necessary radiographs were also carried out. The clinical assessment was carried out as per the criteria suggested by Majeed et al [6]. (Table 1). The radiological assessment was also carried out according to the parameters observed on the plain roentgenograms done at each follow up visit (Table 2).

**Table 1:** (Clinical scoring: Majeed et al)

Patient ability	score
Pain	
Intense, continuous at rest	0 to 5
Intense with activity	10
Tolerable, but limits activity	15
With moderate activity, abolished by rest	20
Mild, intermittent, normal activity	25
Slight, occasional or no pain	30
**Maximum**	**30**

Sitting	
Painful	0 to 4
Painful if prolonged or awkward	6
Uncomfortable	8
Free	10
**Maximum**	**10**

Sexual intercourse	
Painful	0 to 1
Painful if prolonged or awkward	2
Uncomfortable	3
Free	4
**Maximum**	**4**

Walking aids	
Bedridden or almost	0 to 2
Wheelchair	4
Two crutches	6
Two sticks	8
One stick	10
No sticks	12
**Maximum**	**12**

Gait unaided	
Cannot walk or almost	0 to 2
Shuffling small steps	4
Gross limp	6
Moderate limp Slight limp	8 10
Normal	12
**Maximum**	**12**

Walking distance	
Bedridden or few metres	0 to 2
Very limited time and distance	4
Limited with sticks, difficult without	6
prolonged standing possible	
One hour with a stick	8
One hour without sticks, slight pain or limp	10
Normal for age and general condition	12
**Maximum**	**12**
**Functional outcome (total score)**	
Excellent	78 to 80
Good	70 to 77
Fair	60 to 69
Poor	<60

**Table 2:** Radiological outcome scores

Outcome	Residual displacement
Excellent	0-5 mm

Good	6-10 mm

Fair	11-15 mm

Poor	>15 mm

## Results

The study included a total of 19 patients with symphyseal diastasis. There was a single patient with APC (anteroposterior compression) I injury, 5 with APC type III and the rest of the patients had type II APC injury.

The mean follow-up was for 2.9 years (range: 6 months to 4.5 years). Two patients were lost to follow-up during the course of the study: a patient with APC I injury (at 6 months post-injury) and another with APC III injury (61 year old diabetic male who had complications of infection and DVT post-operatively; lost to follow-up at 7 months). The clinical and radiological evaluations of all the patients were carried-out at the last out-patient department visit of these patients. The general profile of our patients, management protocols followed, types of fixation used, complications observed and the respective clinical and radiological scores have been tabulated below (Table 3).

**Table 3:** Patient profile

**S. No**.	Patient profile [Age in years (weight in kg)]	Type of injury	Mechanism of injury	Fixation: single(S) or double(D) plating	Associated conditions	Complications	Late problems	Majeed score [clinical (radiological)]
1	40 (62)	APC I	MVI	Nil				-

2	35 (68)	APC II	CI	Ant (S)	Open injury	Infection		72 (5)

3	40 (75)	APC II	MVI	Ant (D)			Implant failure	62 (8)

4	43 (66)	APC II	PI	Ant (S)				78 (3)

5	54 (60)	APC II	PI	Ant (D)				72 (6)

6	62 (76)	APC II	CI	Ant (S)	Hypotension			64 (10)

7	52 (81)	APC II	MVI	Ant (D)				74 (6)

8	32 (85)	APC II	MCI	Ant (S)			Implant failure with bladder herniati-on	56 (13)

9	46 (72)	APC II	PI	Ant (D)	Bladder injury			72 (6)

10	41 (69)	APC II	MCI	Ant (S)				76 (7)

11	43 (55)	APC II	CI	Ant (D)				66 (10)

12	25 (66)	APC II	PI	Ant (D)			Implant failure	54 (18)

13	20 (72)	APC II	CI	Ant (S)				70 (7)

14	55 (80)	APC II	PI	Ant (S)	Hypotension			66 (14)

15	61 (73)	APC III (Saroiliac joint disruption)	CI	Ant(S) + Post		DVT, Infection		

16	33 (66)	APC III (sacrum fracture)	CI	Ant(S) + Post				66 (10)

17	38 (64)	APC III (Sacroiliac joint disrupti-on)	PI	Ant(S) + Post				62(12)

18	25 (76)	APC III (ilium fracture)	MCI	Ant(D) + Post	Hypotension			72 (8)

19	22 (58)	APC III (ilium fracture)	CI	Ant(S) + Post	Hypotension			74 (6)

MVI = Motor vehicle injury (car/four wheeler), CI = Crush injury, PI = Pedestrian injury, MCI = Motor cycle (or two wheeler) injury.

Among the 13 patients with APC II injuries, the clinical scores were excellent in one (7.6%), good in 6 (46.15%), fair in 4 (30.76%) and poor in 2 (15.38%). Radiological scores were excellent in 2 (15.38%), good in 8 (61.53%), fair in 2 (15.38%) and poor in one patient (7.6%). Among the 5 patients with APC III injuries, there were 2 patients each with good (50%) and fair (50%) clinical scores while one patient was lost on long term follow up. The radiological outcomes were also similar in these.

Among the patients with APC II injury, 7 patients (53.84%) had undergone single symphyseal plating and 6 (46.15%) had double symphyseal plating. In the single symphyseal plating group, outcomes as assessed clinically were excellent in one patient (14.28%), good in three (42.85%), fair in two (28.57%) and poor in one patient (14.28%). The patients with double symphyseal plating had three good (50%), two fair (33.33%) and one poor (16.67%) clinical outcome. Although the data was insufficient for statistical analysis to be performed, there was no obvious difference in the clinical outcomes between single anterior and double perpendicular plating techniques. The radiological outcomes of the two groups were also assessed and compared. There were two excellent (28.57%), three good (42.85%) and two fair (28.57%) results in the group with single plating as against five good (83.33%) and a single (16.67%) poor outcome in the double plating group.

There were 2 patients each with APC Type II (15.38%) and type III (50%) injuries who had hypotension at presentation. All these patients were resuscitated initially with crystalloids and pelvic compression was given using a pelvic binder. A central venous access was also obtained for regular monitoring of central venous pressure and blood transfusion done as required. However, two patients (one each with APC type II and type III injury), could not be stabilised despite the above interventions and external fixator was applied which ultimately arrested the hemorrhage.

There was a single case of associated urethral injury that was managed by immediate supra-pubic cystostomy followed by secondary urethral repair at a later date. Another patient with APC II injury had a Gustilo Anderson grade II open diastasis in which an external fixator was applied at the first stage. Open reduction and internal fixation was done later when the local wound condition permitted.

Postoperative complications included two cases of infection that was evident during the hospital stay of the patients presenting with active sero-purulent discharge at the incision site. The first patient was a 35 year old male who had initially presented with open, type II APC injury. The wound, in this patient, required debridement once on the 13^th ^post-operative day after which the infection settled down satisfactorily. The other patient was a 61 year old obese, diabetic male who had presented with fever on the 6^th ^post-operative day and wound discharge on the 13^th ^post-operative day. The wound failed to heal satisfactorily and needed two more debridements till the 7^th ^post-operative month. The same patient had developed swelling on the right lower limb on the 20^th ^post-operative day that was investigated and diagnosed as a right sided iliofemoral deep venous thrombosis. The swelling subsided subsequently following medical treatment by the concerned experts. However, the patient was lost to follow-up after 7 post-operative months and could not be traced till date.

**Figure 5:**
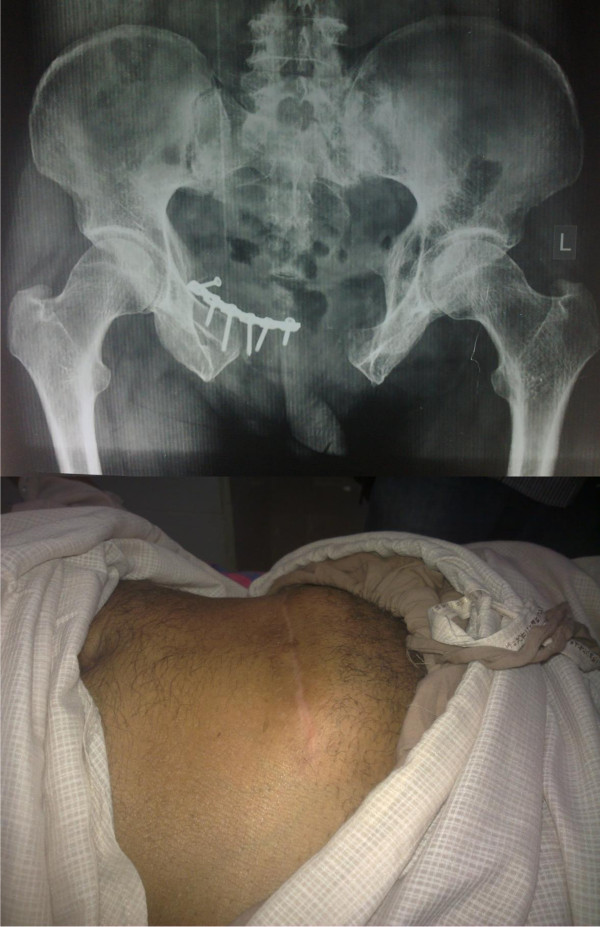
**Implant failure with bladder herniation in one of the patients; radiological and clinical images**.

**Figure 6:**
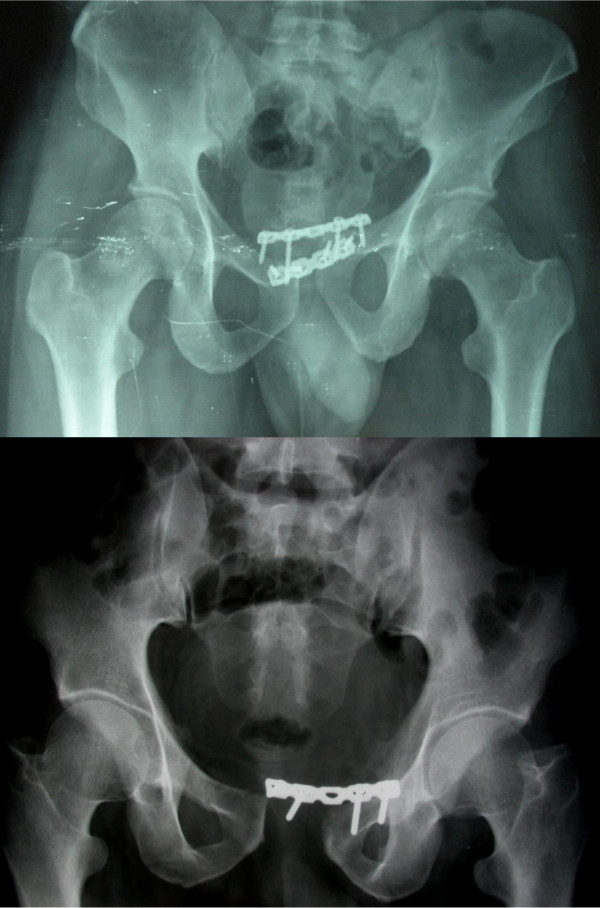
Implant failure in the two other patients showing plate pull out

There were 3 patients with implant failure (Figure 5 and Figure 6) due to plate pull out. The pelvic ring opened up in two of these patients. One of these patients developed urinary bladder herniation from the incision site (Figure 5). All these patients recovered well with implant removal and repeat symphyseal plating. The one patient with bladder herniation required hernia repair by the general surgery team and continues to be asymptomatic at the last follow up after 2 years.

## Discussion

There have been long-standing controversies in classifying the pelvic ring fractures as stable and unstable patterns. Olson has described stable injury as one that withstands the physiological forces incurred with protected weight bearing or bed to chair mobilization without abnormal deformation of the pelvis, until bony or soft tissue healing occurs [1]. The unstable pelvic fractures are fraught with a number of complications and demand timely interventions including adequate resuscitation and appropriate, stable fixation to ameliorate the morbidity and mortality associated with these injuries [7].

The patients included in our study had the antero-posterior compression type of injury, most common of which are the APC type II disruptions. These injuries predominantly involve the young male population and typically follow high energy road traffic accidents. As already emphasised, the earliest interventions that can save lives in these situations are resuscitation and control and management of hemorrhage [8]. The importance of the radiological investigations especially computerised axial tomography in the surgical planning cannot be understated, although resuscitation and patient stabilisation must take precedence over these diagnostic procedures.

Although the surgical management of the antero-posterior compression injuries has not been straight-forward[9-12] and fraught with a number of controversies, there is a general consensus on the need for adequate surgical fixation and stabilisation when the symphyseal gap exceeds 2.5 cm. Early non-invasive stabilisation using a pelvic binder or pelvic sling to provide circumferential compression, or emergent, mini-invasive, compression techniques using the external fixators or C-Clamp (Ganz et al) may be necessary to arrest life threatening bleeding. Symphysis contact by these external appliances may be achieved by delivering forces as high as 177 ± 44 N and 180 ± 50 N for reduction of the partially stable and unstable pelves, respectively. The ideal management is, however, provided by stable, internal fixation only [12]. There again, the controversy arises on the adequacy of single symphyseal plating, the need for double (perpendicularly placed) symphyseal plates, the ideal placement site of the plates (superior or anterior symphyseal surfaces), the types of plates used (reconstruction or low contact dynamic compression plates), the situations that need additional posterior pelvic stabilization, and so on. Although approach to the pubic symphysis using Pfannensteil incision is well-established and universally employed, a few authors have suggested the feasibility of minimally invasive techniques with indirect reduction and percutaneous fixation using multiple screws [13-15].

Classification systems have been considered the key-stone in deciding the management protocols in pelvic fractures [5]. Although, the need for an additional posterior ring stabilisation (apart from symphyseal plating) to negate the vertical instability at sacro-iliac joint in type III APC injuries has been well acclaimed, a similar fixation in type II injuries has been an issue of debate over the past few decades. The anterior sacro-iliac ligament gets violated in all cases where the pubic symphysis is displaced more than 2.5 cm. Kapandji [16] has proposed that a small amount of nutation (nodding) movements occurs at the sacro-iliac joints with physiological weight bearing in these conditions (APC II). These movements tend to get transmitted anteriorly to the pubic symphysis. Multiple forms of symphyseal plate fixations like 4-hole dynamic compression plates, special angled plates, long plates and double-plate fixation have all been tried in type II APC injuries [17-19]. Single, anteriorly placed symphyseal plate provides a greater resistance to external rotation forces than superiorly placed plates in these antero-posterior compression injuries and is biomechanically, a more rigid fixation [16].

Lange et al [20] had used the anterior 2-hole plate fixation in symphyseal diastasis. The symphyseal double plate fixation (combination of anterior and superior symphyseal plates) provides the most rigid fixation of all; however, the procedure requires considerable dissection, expertise and time and may be associated with significant blood loss. The anterior 2-hole plate is a much less rigid fixation and helps in accommodating the physiological motion at the symphysis, yet adequately resisting the tensile stresses across the symphysis without loss of reduction. The soft tissue collar and tether provided by the inguinal ligament are not disrupted by the minimal dissection required for two-hole plate fixation.

Simonian et al [21,22] had concluded that combined anterior and posterior fixation was optimal for APC type II injuries. Dujardin et al [23] also reported a decrease in the micromotions at the SI joint in these injuries when combining anterior plate fixation with sacroiliac fixation compared with isolated anterior plate fixation. MacAvoy et al [24] on the other hand suggested that single anterior plating of the pubic symphysis had similar biomechanical properties to two plates in pelvis with isolated rotational instability. They reported no difference between single and double plate fixation of the symphysis. Tile et al [25] had also concluded single anterior symphyseal plating as the ideal and sufficient fixation for APC injuries with a displacement of the posterior ring of less than 1 cm (rotationally unstable but vertically stable pelvic ring).

We have evaluated the clinical and radiological outcomes in our patients to assess the influence of multiple variables on the long term results. The presence of posterior ring injuries (APC III vs. APC II) is known to have a significant negative impact on the long term outcome although in our series the results were comparable when the posterior ring disruptions were adequately stabilized simultaneously. Almost half of the patients with APC type III injuries in our series presented with significant blood loss and hypotension. The urethral injury, although seen in only one of our patients, commonly accompanies such injuries and occurs as a result of shear forces at the junction of the prostatic and membranous urethra. Bladder/urethral injuries are also known rare surgical complications that occur during operative fixation of the symphyseal diastasis following inadvertent invasion of the viscus by inexperienced surgeons. There was a single case of post-injury urethral rupture (5.2%) in our series. The management of these genito-urinary injuries has been controversial with one school of surgeons supporting a supra-pubic cystostomy followed by a secondary repair of the urethral stricture and another school supporting supra-pubic cystostomy and primary urethral repair at the same sitting. We had performed an immediate supra-pubic cystostomy followed by the secondary urethral repair by an expert urologist.

One of the patients in our series developed urinary bladder herniation postoperatively. This complication, most probably results from an inadequate reduction of the diastasis or improper repair of the rectus sheath. We believe that in cases with marked disruption of the symphysis, avulsion of one head of the rectus abdominis is a common finding and there is no need to detach the rectus abdominis from the other side. Further, transverse sectioning of the rectus abdominis should be avoided as this impairs subsequent repair and healing of the abdominal wall. A careful surgical dissection and a meticulous repair go a long way in preventing soft tissue problems like bladder herniation in long run.

Although we used double symphyseal plating in one of our patients with Type III injury, we found single symphyseal plate along with posterior fixation to be adequate in stabilising such fractures. Some authors have recommended double symphyseal plating to be more stable fixation modality in these injuries with biplanar instability [20,26,27]. However, from our experience, we believe that a single plate provides an equally stable construct when combined with posterior ring fixation. Some authors have also suggested double symphyseal plating as the lone stabilisation procedure in APC III. On the contrary we believe that, if the posterior ring disruption is neglected, such a construct leads to a more compromised stability biomechanically.

Although our sample size was small for appropriate statistical tests to be done, we believe that the addition of the superior symphyseal plate does not add to the stability offered by a single anterior plate (contrary to the claim in the literature that the double plating technique offers greater rigidity). We reported 3 cases of implant failure in our series. This could have been partly due to inadequate reduction of the diastasis and party due to improper repair of the rectus insetion. We also believe intactness of the rectus abdominis insertion significantly adds to the stability of the constructs and this should be ensured whenever possible.

Our study had a few potential limitations. We had not used any patient validated scores (SF 12 or SF 36) or the assessment of the Activities of Daily Living (ADL) to evaluate the outcome. Nevertheless we believe that the clinical and radiological scores used by us for follow up assessment give us a fair idea about the functional outcome in our patients. The smaller sample size in our study also prohibited application of tests of significance. Nevertheless we share our experience in management of these devastating injuries.

To conclude, we believe that there is no gross dissimilarity in the outcomes between isolated anterior and combined symphyseal (perpendicular) plating techniques in APC II injuries. Single anterior symphyseal plating along with posterior pelvic ring stabilisation provides a stable fixation in type III APC injuries. We also believe that the amount of reduction achieved (gap less than 1 cm) is an important, independent variable in determing the long term outcome. Limited dissection and preservation of intactness of rectus sheath go a long way in avoiding post-operative complications and ensuring a satisfactory long term outcome.

## Conflict of interests

The authors declare that they have no competing interests.

## Authors' contributions

KB and VK1 reviewed the literature and wrote the paper. SA and RKS were main operating surgeons in the whole series and critically reviewed the paper. KB, VK2 and DM maintained all the records of the patients and followed them. All the authors read and approved the final manuscript
